# Environmental Challenges in Southern Brazil: Impacts of Pollution and Extreme Weather Events on Biodiversity and Human Health

**DOI:** 10.3390/ijerph22020305

**Published:** 2025-02-18

**Authors:** Joel Henrique Ellwanger, Marina Ziliotto, Bruna Kulmann-Leal, José Artur Bogo Chies

**Affiliations:** Laboratory of Immunobiology and Immunogenetics, Postgraduate Program in Genetics and Molecular Biology (PPGBM), Department of Genetics, Institute of Biosciences, Universidade Federal do Rio Grande do Sul (UFRGS), Porto Alegre 91501-970, Rio Grande do Sul, Brazil; joel.ellwanger@gmail.com (J.H.E.); marinaztto@gmail.com (M.Z.); bruna.k.leal@gmail.com (B.K.-L.)

**Keywords:** Brazil, biodiversity, climate change, extreme weather events, particulate matter, pesticide, plastics, pollution, potentially toxic elements, Rio Grande do Sul

## Abstract

The Amazon rainforest plays a fundamental role in regulating the global climate and therefore receives special attention when Brazilian environmental issues gain prominence on the global stage. However, other Brazilian biomes, such as the Pampa and the Atlantic Forest in southern Brazil, have been facing significant environmental challenges, either independently or under the influence of ecological changes observed in the Amazon region. The state of Rio Grande do Sul is located in the extreme south of Brazil and in 2024 was hit by major rainfalls that caused devastating floods. The Pampa is a non-forest biome found in Brazil only in Rio Grande do Sul. This biome is seriously threatened by loss of vegetation cover and many classes of pollutants, including pesticides and plastics. Mining ventures are also important sources of soil, water and air pollution by potentially toxic elements in Rio Grande do Sul, threatening both the Pampa and the Atlantic Forest. Furthermore, southern Brazil is often affected by pollution caused by smoke coming from fires observed in distant biomes such as the Pantanal and the Amazon. Considering the significant environmental challenges observed in southern Brazil, this article revisits the historical participation of Rio Grande do Sul in Brazilian environmentalism and highlights the main environmental challenges currently observed in the state, followed by an in-depth analysis of the effects of pollution and extreme weather events on biodiversity and human health in the region. This review encompassed specifically the following categories of pollutants: potentially toxic elements (e.g., arsenic, cadmium, chromium, cobalt, copper, lead, mercury, titanium), air pollutants, plastics, and pesticides. Pathogen-related pollution in the context of extreme weather events is also addressed. This article emphasizes the critical importance of often-overlooked biomes in Brazilian conservation efforts, such as the Pampa biome, while also underscoring the interconnectedness of climate change, pollution, their shared influence on human well-being and ecological balance, using Rio Grande do Sul as a case study.

## 1. Introduction: A Brief Overview of Eco-Social Aspects of Rio Grande do Sul

**Figure 1:**
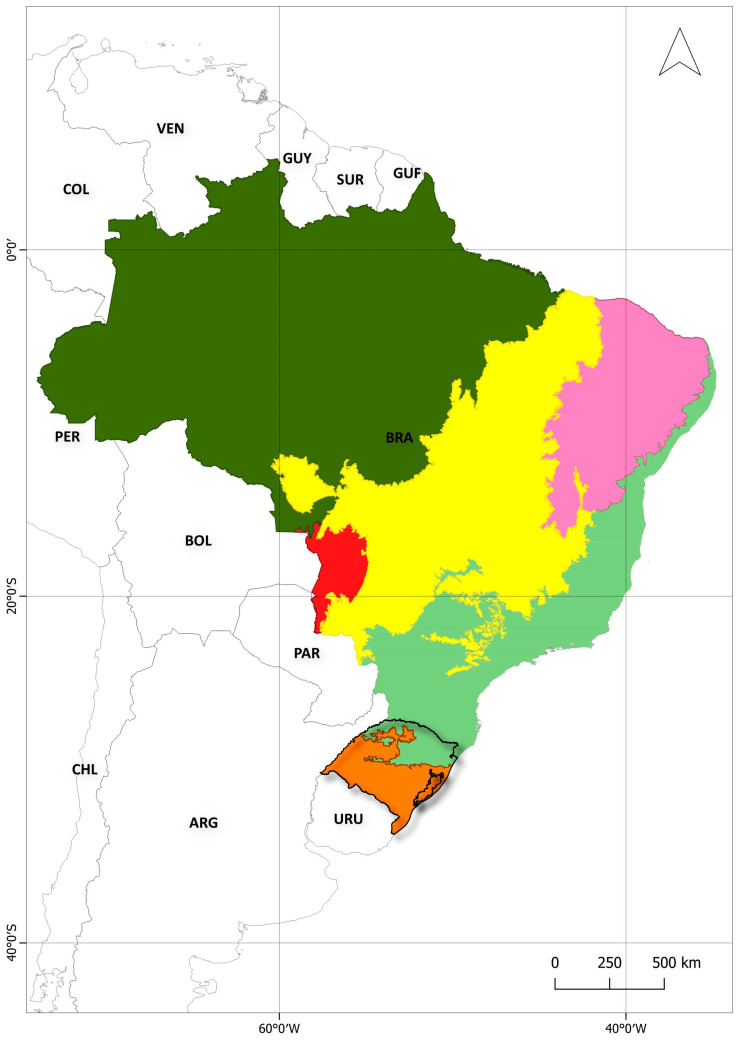
Brazil’s map showing the distribution of the terrestrial Brazilian biomes. Rio Grande do Sul state is highlighted on the edge in bold. In orange: coverage of the Pampa biome. In light green: coverage of the Atlantic Forest biome. In yellow: coverage of the Cerrado biome. In red: coverage of the Pantanal biome. In pink: coverage of the Caatinga biome. In dark green: coverage of the Amazon biome. Brazil is located in Latin America and shares borders with the following countries: French Guiana (GUF), Suriname (SUR), Guyana (GUY), Venezuela (VEN), Colombia (COL), Peru (PER), Bolivia (BOL), Paraguay (PAR), Argentina (ARG), and Uruguay (URU), as shown on the map. Chile (CHL) is also visible. Coordinates obtained using SIRGAS2000.

**Figure 2:**
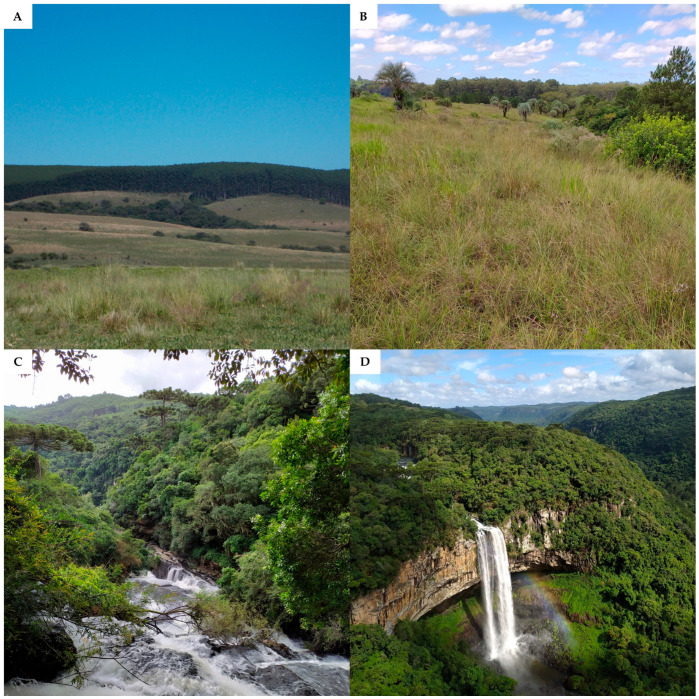
Representative images of Rio Grande do Sul landscapes. (**A**) Pampa biome in São Gabriel City, showing predominant grassy vegetation in the foreground and forestry activity in the background, one of the biggest current threats to the Pampa (photo credit: Alexandre Copês). (**B**) Ecotone zone near Porto Alegre City, showing the transition between the Pampa and Atlantic Forest biomes (photo credit: Joel H. Ellwanger). (**C**,**D**) Mountainous region of Rio Grande do Sul, Canela City, showing mixed ombrophilous forest belonging to the Atlantic Forest biome (photo credits: Joel H. Ellwanger).

**Figure 3:**
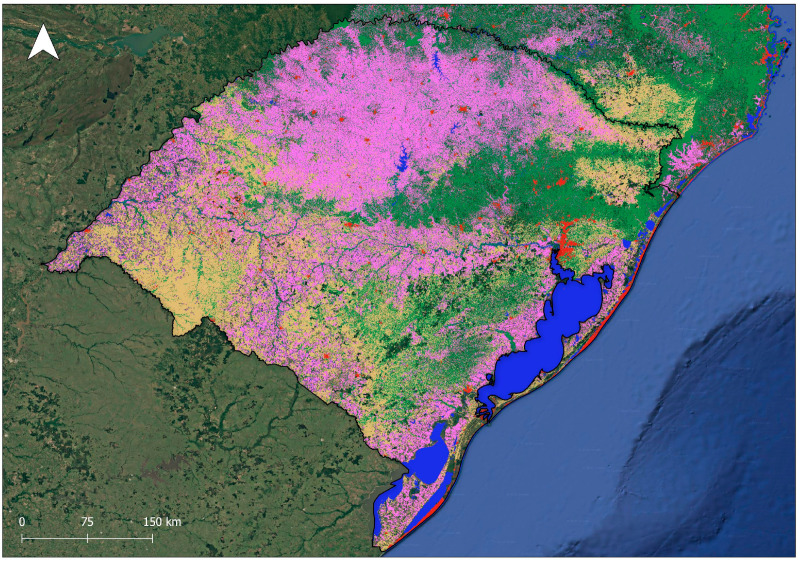
Map of Rio Grande do Sul. The state border is highlighted on the edge in bold. In green: distribution of forests. In yellow: distribution of grasslands. In pink: distribution of agriculture. In red: distribution of areas without vegetation (composed of urban areas, mining, beaches, dunes, sand spots, and other regions without vegetation). In blue: water bodies. Images from Google Satellite and data from MapBiomas.

Rio Grande do Sul is a Brazilian state located in the extreme south of Brazil, bordering Argentina and Uruguay [1], featuring a 620 km coastal plain along the Atlantic Ocean, from the mouth of the Mampituba River (Torres City, in the north) to the mouth of the Chuí Stream (in the south) [2,3]. Figure 1 shows the Brazilian terrestrial biomes, highlighting the location of Rio Grande do Sul on the Brazilian map. Two biomes occur in Rio Grande do Sul: the Pampa, which covers 62% of the state territory and is characterized mainly by grassland vegetation [4], and the Atlantic Forest, a forest biome of extreme ecological importance [1]. The transition area between the Pampa and the Atlantic Forest (the ecotone zone) and the contact between different phytoecological regions (areas of ecological tension) create ecological complexity and favor the occurrence of rich biodiversity in the state [1,5,6,7,8], with multiple endemic plant species [5]. Figure 2 shows some representative images of Rio Grande do Sul landscapes. Alarmingly, Rio Grande do Sul’s vegetation is highly threatened by agricultural activities, forestry activities, and the expansion of urban settings, among other anthropic pressures [1,4]. Figure 3 displays a map of Rio Grande do Sul’s vegetation cover and human-altered areas, based on data from MapBiomas [9].

The oldest human settlers in Rio Grande do Sul date back to 13,000 and 8500 years before the present [10]. The Indigenous populations of the Charrua, Kaingang, Minuano, Xokleng, and Guarani are the main native peoples of Rio Grande do Sul, which received large numbers of immigrants from Europe especially between the 19th century and the first half of the 20th century, although the European presence in the state dates back to the 16th century [10,11,12]. As a result, a significant proportion of the population in Rio Grande do Sul today has European ancestry, showing traits that reflect a strong genetic admixture from European, African, and Indigenous American peoples [13].

With a population of 10,882,965 people (2022 data), 497 cities, and a total territory of 281,707.151 km^2^, Rio Grande do Sul is one of the most developed states in Brazil when the Human Development Index is considered (score of 0.771—high human development according to 2021 data), occupying 5th position in the ranking of Brazilian states [14]. In addition to having a diversified industrial hub and a thriving services sector, Rio Grande do Sul’s economy is strongly tied to agricultural production [15].

Currently, social, economic, political, and climate issues threaten both human and environmental health in Rio Grande do Sul, a state with a strong tradition of environmental protection. These contradictions and challenges will be detailed and discussed in this narrative review.

## 2. Objective and Methodological Notes

The purpose of this article is to provide an update on the main environmental challenges faced by southern Brazil, using Rio Grande do Sul as a case study, focusing on the impacts of pollution and extreme weather events on biodiversity and human health. This article is a narrative review based on documents accessed through the databases PubMed [16], Scientific Electronic Library Online—SciELO [17], and Google Scholar [18]. Searches were performed in September 2024 in these three databases using the following terms, in both English and Portuguese: “Rio Grande do Sul” + “pollution” in combination with “atmospheric” or “air” or “soil” or “water” or “smoke” “plastic” or “microplastic” or “potentially toxic element” or “heavy metal” or “metal” or “mining” or “pesticide” or “agrochemical” or “extreme weather event” or “climate change” or “flood” or “drought” or “fire”.

The articles/documents were selected in a non-systematic manner based on the relevance of the results and information, with no restriction on the year of publication. Although these criteria were the starting point for the selection of the articles/documents cited in the review, other sources cited in the list of references of the documents and articles that make up the digital library of the authors regarding ecological and historical aspects of Rio Grande do Sul were also used to complement the sources of this review. Relevant online sources to contextualize the topics discussed in the review were also used. We highlight that this is not an exhaustive review, but rather an update and synthesis on the different topics covered in the article.

It is important to emphasize that Rio Grande do Sul has a historical role in Brazilian environmental protection. Therefore, before presenting the environmental challenges currently observed in the state, we revisit some historical milestones of environmental activism in Rio Grande do Sul. In brief, this review is structured as follows: First, some historical landmarks for environmentalism in Rio Grande do Sul are presented (Section 3). Next, the main pollution-related issues in Rio Grande do Sul are detailed (Section 4), including information on recent extreme weather events observed in the state (Section 5). Following this, the impacts of the main groups of pollutants on human health and biodiversity are detailed and discussed, including potentially toxic elements (Section 6), air pollutants (Section 7), plastic pollutants (Section 8), and pesticides (Section 9). How extreme weather events exacerbate the effects of pollution on human health and biodiversity in Rio Grande do Sul are then discussed in Section 10, with a focus on pathogen pollution. Finally, some perspectives (Section 11) and conclusions (Section 12) are presented.

## 3. Revisiting the Historical Participation of Rio Grande do Sul in Brazilian Environmentalism

Throughout the Brazilian “economic miracle” that occurred between the late 1960s and 1973 during the military (dictatorial) regime, pollution and environmental degradation were considered acceptable, as a “price to be paid” for the high economic development observed in that period. Industrial development unrelated to environmental concerns quickly began to harm the health of populations affected by pollution in large cities in several Brazilian states, including Rio Grande do Sul [19]. Brazilian environmentalism emerged from the 1970s onwards as an educational effort regarding Brazil’s environmental problems, and as a movement to oppose the vision of exploiting natural resources at any cost, questioning the socio-economic development models in force at that time [20]. Rio Grande do Sul played a prominent role in this movement, with important actions during and before the 1970s.

In 1955, the environmentalist Henrique Luiz Roessler (1896–1963) created the Nature Protection Union (União Protetora da Natureza—UPN) in São Leopoldo City, the first non-governmental nature protection entity in Rio Grande do Sul. Another prominent figure in the environmental movement was José Lutzenberger (1926–2002), one of the first Brazilian environmentalists to gain international prominence for denouncing environmental degradation in Brazil. He was born and worked many years of his life in Rio Grande do Sul, and played a leading role in founding, in 1971, the Gaucho Association for the Protection of the Natural Environment (Associação Gaúcha de Proteção ao Ambiente Natural—AGAPAN), one of the most important environmental entities in Brazil, headquartered in Porto Alegre City, the capital of Rio Grande do Sul [21,22,23].

Many other milestones in Brazilian environmentalism are linked to Rio Grande do Sul. In the 1970s, civil society action targeting the activities of the Borregaard Cellulose Industry, of Norwegian origin but located in Guaíba City, gained attention due to the company’s significant contribution to pollution in the region [19,24]. In 1982, the State Law on Pesticides (Lei Estadual dos Agrotóxicos No. 7747/82) was approved, an important pioneering initiative to regulate the use of these chemical agents in Rio Grande do Sul [22,23]. More recently, the efforts of environmentalists have been focused on mining projects with high polluting potential in the state [24].

Porto Alegre has also hosted the Ecological Farmers’ Fair (Feira dos Agricultores Ecologistas—FAE) since 1989 [23,25] and the Bom Fim Ecological Fair (Feira Ecológica do Bom Fim—FEBF) since 1991 [26], two of the most important and long-lasting initiatives for selling agroecological products in Brazil, thus contributing to limiting pesticide pollution. Currently, there are a total of eight officially recognized agroecological fairs in the city of Porto Alegre alone [27].

Initiatives like these indicate a desire on the part of the population of Rio Grande do Sul to counteract the growing environmental degradation activities in the state during the last decades of the 20th century. In this sense, along with the nature protection activities of other prominent environmentalists, such as Balduino Rambo (1905–1961), Augusto Carneiro (1922–2013), Hilda Zimmermann (1923–2012), and Flávio Lewgoy (1926–2015), the actions of Roessler and Lutzenberger made it clear that Rio Grande do Sul is facing serious environmental challenges, especially since mid-20th century, including pollution triggered by vehicles, poor sanitation systems, excessive use of pesticides, vegetation fires, and highly polluting industries [21,22,23].

The situation concerning pollution has not improved significantly in recent years. Gasoline- and diesel-powered vehicles are the main causes of vehicle pollution in the metropolitan region of Porto Alegre [28]. In this region, vehicular pollution is added to strong industrial activity, coal combustion, and wood burning (i.e., to produce charcoal). Together, these factors release a significant quantity of carbon monoxide, polycyclic aromatic hydrocarbons (PAHs), and other pollutants into Rio Grande do Sul’s atmosphere [29,30]. Pollution in the state goes beyond that detected by sensitive pollutant measuring equipment. A study carried out in Rio Grande City, located on the southern coast of the state, indicated that 68% of the population notices pollution in their daily lives, with deleterious effects on health and well-being [31].

## 4. Main Pollution-Related Issues in Rio Grande do Sul

The Brazilian colonization process left pollution marks that have lasted until the present day in Rio Grande do Sul. Notably, in the urban region of Rio Grande City, land reclamation fills established in the Brazilian colonial period are sources of pollution from mercury (Hg) and other potentially toxic elements (PTEs), adding to current sources of pollution. The processing of animal skins from the 18th century onwards was common in Rio Grande and used large amounts of Hg, and this is probably the main cause of the high levels of Hg pollution found in the city [32].

More recent historical events also show a significant pollution legacy. Urbanization in Rio Grande do Sul and other Brazilian states occurred simultaneously with the industrialization process. This resulted in many residential areas being established very close to sources of industrial pollution. Furthermore, areas previously occupied by industries, and potentially contaminated, were later occupied by human habitation, exposing humans and animals to residual industrial pollutants [32]. In Rio Grande do Sul, cities with the highest Industrial GDP are precisely those with a greater propensity for pollution [33].

Chemical colonialism is strongly observed in southern Brazil [34]. European countries export huge quantities of pesticides to Brazil, many of which are banned in the European Union, for the production of commodities such as soybeans in Brazil, exposing the Brazilian population and biodiversity to the toxic effects of pesticides, while at the same time apparently sparing European populations. Of the ten most widely used pesticides in Brazil, five are banned in the European Union: mancozeb, atrazine, acephate, chlorothalonil, and chlorpyrifos [34]. Rio Grande do Sul is one of the largest consumers of pesticides in Brazil due to its leading role in agricultural production. As a result, the human and animal populations in the state suffer significantly from the harmful effects of pesticides due to the contamination of aquatic and terrestrial ecosystems with these chemicals [35,36].

In the Pampa biome, urban expansion, agriculture, livestock farming, forestry activities and other anthropic uses currently occupy approximately half of the biome, being the main causes of the degradation of this fragile grassland ecosystem [4,36,37]. Both livestock farming and loss of vegetation cover due to cash crop plantations contribute significantly to atmospheric pollution by greenhouse gases [38]. Also, mining and industrial activities with high polluting potential, the disposal of solid waste as well as domestic and industrial effluents into the environment, among other anthropogenic problems, coupled with deficient environmental monitoring, cause contamination of the air, soil, and water of Rio Grande do Sul’s ecosystems with the most diverse classes of pollutants [37,39,40,41,42,43,44,45].

Marine and freshwater ecosystems in the Rio Grande do Sul are contaminated with macro and microplastics, significantly affecting fauna through accidental ingestion of plastics and the deleterious effects of plastic-derived toxic chemicals [46,47,48,49]. The wetlands of Rio Grande do Sul suffer from a variety of anthropogenic impacts, such as garbage dumping and contamination with domestic sewage and chemical products (pesticides, among others), affecting the fauna and flora of these sensitive, rich in biodiversity, and highly biologically productive ecosystems [50].

Porto Alegre has a deficient sewage treatment system, which contributes to the pollution of the Dilúvio Stream, which runs through the city and flows into the Guaíba Lake, one of the most important aquatic ecosystems in the state. Despite its great importance for the metropolitan region of Porto Alegre as a leisure area, transportation of passengers and products, as well as a water supply for the population, the Guaíba Lake is polluted by several classes of pollutants [51,52].

Porto Alegre’s metropolitan region is also experiencing a boom in real estate development [53]. For this reason, we highlight that construction activities are directly and indirectly a significant source of pollution in Rio Grande do Sul, with pollutants ranging from CO_2_ emissions associated with the use of concrete (indirect pollution) to construction waste (direct pollution), which ranges from rubble to paints and solvents. Irregular disposal of this waste is even observed in water springs and environmentally protected areas. In addition to damaging ecosystems, the disposal of solid waste such as construction waste into the environment causes visual pollution [54,55].

**Figure 4:**
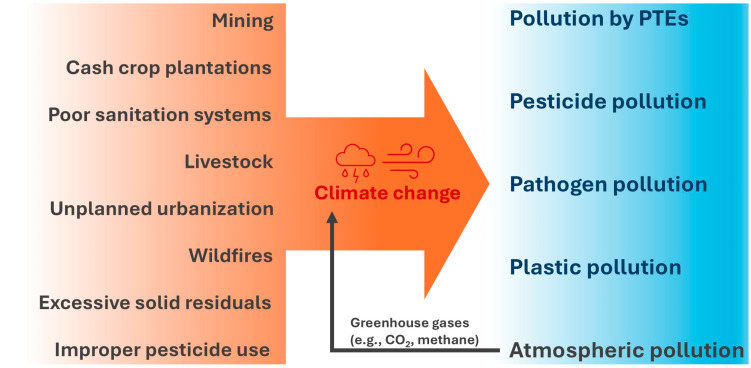
Main anthropogenic activities and pollution classes observed in Rio Grande do Sul. Atmospheric pollution fuels climate change, which exacerbates the impacts of other pollution classes. PTEs—potentially toxic elements. CO_2_—carbon dioxide.

In summary, Rio Grande do Sul is currently impacted by a series of different pollutants that reflect an accumulation of varied polluting sources from the past combined with polluting sources from the present. Other environmental challenges, such as climate change, tend to exacerbate this problem (Figure 4).

## 5. Recent Extreme Weather Events Observed in Rio Grande do Sul

Brazil has been facing diverse consequences of climate change, such as floods, landslides, droughts, wildfires, and heat waves [56]. Global climate change is already evident in Rio Grande do Sul through significant thermal and rainfall changes. Specifically, increases in temperature, precipitation, and humidity have been observed between 1961 and 2004 [57]. From 1972 to 2015, 132 climate disasters were recorded in the mid coast area of Rio Grande do Sul [58]. Associated with meteorological phenomena frequently observed in the state, such as mesoscale convective complexes, climate change may intensify heavy rains, hailstorms, landslides, floods, and strong winds [58,59]. Of note, severe droughts have affected southern Brazil in recent years, with significant impacts in Rio Grande do Sul in 2019 and 2020 [60,61]. Climate anomalies such as El Niño, a period with higher-than-expected rainfall, may cause erosion and other important changes in the soil of southern Brazil [62]. This can exacerbate the effects of climate change, such as through increased risks of landslides and flashfloods. Such a climate scenario is highly alarming since climate projections for the Rio Grande do Sul indicate an increase in the frequency of extreme weather events in the coming decades due to climate change [58].

**Figure 5:**
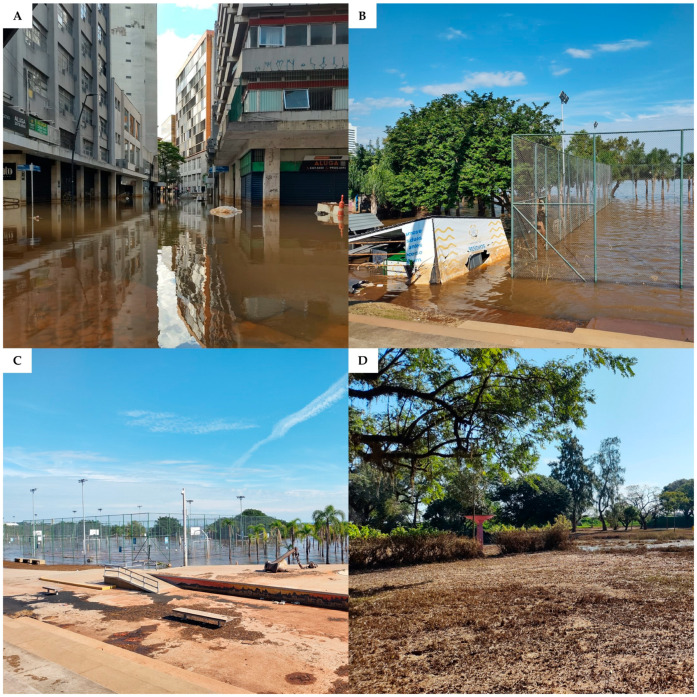
Rio Grande do Sul 2024 flood. (**A**) Central region of Porto Alegre City. (**B**,**C**) The Guaíba Lake shore. (**D**) Vegetation on the Marinha Park shore (Porto Alegre) severely impacted after being submerged for several days. (Photo credits: Alexandre Copês).

Extreme rainfall and floods affected the Taquari-Antas Basin in September 2023, causing dozens of deaths and placing several cities in a state of calamity [63]. In 2024, catastrophic floods affected 96% of Rio Grande do Sul cities between April and May, causing 183 deaths and affecting more than 2.4 million people. Regions of some major cities, such as Porto Alegre (Figure 5), remained flooded for a month [64,65].

The floods recorded in Rio Grande do Sul in 2024 were the result of a combination of El Niño and climate change (i.e., warmer than normal sea surface waters) [66,67] and had their ecological and social effects intensified by climate change denial, unplanned urbanization, and deficient climate disaster prevention [66,68]. The Rio Grande do Sul 2024 mega flood was considered the biggest climate disaster ever observed in Brazil due to the huge number of people affected and extensive infrastructure damage [68].

Climate change will impact the water and terrestrial ecosystems of both the Atlantic Forest and Pampa, with consequences such as habitat reduction, changes in species composition, and biodiversity loss [69,70]. Therefore, the combined effects of climate change and pollution are a major challenge for the Brazilian environmental agenda [71].

## 6. Pollution by Potentially Toxic Elements

### 6.1. Impacts on Humans

Potentially toxic elements (PTEs) are natural components of the Earth found mainly in soil and rocks, such as lead (Pb), cadmium (Cd), zinc (Zn), copper (Cu), arsenic (As), chromium (Cr), nickel (Ni), mercury (Hg), manganese (Mn), and cobalt (Co), among others [72]. Some PTEs in small (trace) amounts are important for the proper functioning of biological systems, such as selenium (Se), a key element in the antioxidant enzyme system. However, large amounts of this element are toxic to humans [73]. Therefore, Se is potentially toxic for humans, depending on its quantity. Some other PTEs, such as Pb, are toxic to humans in any amount [74,75]. Pollution by PTEs impacts human health mainly through environmental exposure and the ingestion of contaminated food [76]. In humans, PTEs can trigger oxidative stress, neurotoxicity, nephrotoxicity, hepatotoxicity, skin toxicity, and cardiovascular toxicity [77].

Rio Grande do Sul is the main wine-producing region in Brazil. However, the region’s humid climate contributes to the proliferation of fungi in the vines [78,79]. Therefore, high doses of fungicides composed of Cu and Zn are used in grape production [78]. In addition to environmental contamination, grape-derived products may contain high doses of PTEs, representing a risk of food-related contamination [79,80,81].

Air pollutants can also carry multiple PTEs. Using lichens *Parmotrema tinctorum* as bioindicators, Koch et al. [82] evidenced air pollution by multiple PTEs, mainly Cu, Zn, iron (Fe), Cr and Ni, in several cities in the northeast region of the state (i.e., Esteio, Triunfo, Charqueadas, Montenegro, Santo Antônio da Patrulha, Caraá, and Maquiné). These cities have major importance for Rio Grande do Sul’s economy, hosting many industries (Triunfo, Esteio, Charqueadas, and Montenegro), a petrochemical complex (Triunfo), and an important agricultural area of family farming (Santo Antônio da Patrulha, Maquiné and Caraá) [82]. In addition, a greater atmospheric presence of PTEs was observed in the region of Caxias do Sul, a large metal–mechanical hub in Brazil, when compared to a rural area [83]. The diversity of economic activities and related impacts on ecosystems can explain the pervasive atmospheric pollution by PTEs, which can pose significant risks to human health, especially when these elements bioaccumulate in human tissues.

### 6.2. Impacts on Biodiversity

Beyond human health, pollution by PTEs has major impacts on the environment and biodiversity. The use of Cu-based fungicides on vines increases the Cu content in the soil, modifying its distribution and desorption in the soil profile. Analyses of Cu deposition in surface soil showed that the use of fungicides in vineyards in the Serra Gaúcha (mountainous region of Rio Grande do Sul) considerably increases Cu pollution in soil [84,85]. Humidity and rainfall not only increase the need for fungicide use, as mentioned previously, but can also facilitate the penetration of Cu into the soil and the pollution of deeper bodies of water. Furthermore, an artisanal Cu-based mixture commonly used in the Rio Grande do Sul’s agriculture, popularly known as the Bordeaux mixture, can contain, besides Cu, other PTEs in its composition, increasing the pollution risk by multiple PTEs [85]. In other words, it is important to consider that the deleterious effects of PTEs on biological systems generally come from multiple combined PTEs, which can amplify the toxic effects of these elements.

The pollution of urban soils with PTEs can act as an important source of damage to biodiversity, and the permanence of PTEs in the environment and the deposition of chemical compounds by human activities leads to the accumulation of these elements in the food chain [86]. The city of Rio Grande is the oldest in the state and its industrial development took place in the 19th century. Geochemical mapping of the industrial and residential sectors of the region showed regions of anomalous presence of Hg, Cu, Zn, Fe, Ni, Cr, and Pb. These regions corresponded to the historical industrial distribution of the city [86]. Furthermore, the Patos Lagoon (Laguna dos Patos), which receives inflows from the city of Rio Grande, presented excess Cu and Pb, which are directly related to the industrial activities in the region [87].

Soil pollution by PTEs has a detrimental impact on the soil environment and its ecology, leading to a reduction in vegetation cover and biomass. The soil’s microbial community (e.g., bacterial and fungal populations) is particularly affected, with a notable decline in microbial diversity [88]. Consequently, the presence of PTEs in soil induces alterations in biogeochemical cycles, ultimately resulting in a decrease in soil fertility and its potential for sustainable use [88,89].

There are some data available concerning Porto Alegre’s pollution by PTEs in the canalized section of the Dilúvio Stream [90], and in the Guaíba Lake, which supplies the city’s population with drinking water [91]. Worryingly, the Guaíba Lake showed pollution by Pb, Cu, Cr, and Ni [91]. The pollution of the waters of the urban region is linked to anthropogenic activities, the deposition of untreated sewage in the network, and the inflow of water from other streams that connect to the lake [91]. The use of landfills in Porto Alegre is also a potential anthropogenic activity responsible for water pollution by PTEs. In this sense, analyses of a landfill on the northern coast of the state revealed the influence of landfill leachate on the quality of groundwater and surface water in the study area [92].

The pollution and bioaccumulation of PTEs represent significant risks to aquatic biota. Many of these pollutants are highly soluble and exchangeable within the aquatic environment, which enhances their biological availability and disrupts biogeochemical cycles [93]. These alterations can adversely affect a range of organisms within the ecosystem, including fish, benthic fauna, and zooplankton, even at low levels of exposure [93]. As benthic organisms become contaminated, the entire food chain is affected, leading to cascading effects that include the loss of biodiversity and a decline in environmental quality [94].

Industrial activities are also responsible for pollution by PTEs in Rio Grande do Sul’s rivers. Industrial, agricultural, and urbanization activities were associated with increased concentrations of PTEs (i.e., Cr, Cu, and Zn) in the Sinos River (Rio dos Sinos) [95], Jacuí Delta (Delta do Jacuí) [i.e., Aluminum (Al) and Cd) [96], and Tramandaí River (Rio Tramandaí) [i.e., Potassium (K), Titanium (Ti), Mn, and Fe] [97]. Sediment analyses at sites close to mining activities demonstrated greater deposition of PTEs [98,99]. Analyses of water bodies and soil near tanning industrial regions, a traditional economic activity in the region, also showed high contamination by PTEs such as Cr and Pb, which are used in the leather production process [100,101,102]. Mining and leather production are recognized as highly polluting activities, contributing significantly to the deposition of PTEs in the environment [103,104]. Of note, the most common elements discarded from these anthropogenic activities generate bioaccumulation and have several adverse effects on ecosystems [76].

The elements Cd, Pb, Cr, and Ni were identified in owls from the north of the state, which are considered bioindicator animals. The presence of high doses of Cr found in these samples indicates the bioaccumulation of toxic elements [105]. Even at low concentrations, the accumulation of these elements can lead to chronic impacts on various wildlife species [106]. Furthermore, the combination of these discarded PTEs negatively affects the growth of plants, algae, and microorganisms exposed to them, while also contributing to the degradation of both soil and aquatic environments [104,106].

Agricultural and human activities in the watershed area of the Caldeirão Steam (Arroio Caldeirão), in the northwest of the region, showed a positive correlation with the increases in the concentrations of Cu, Ni, As, and Cr. The main sources of pollution in the basin were swine farms, stables, human habitation areas, poorly managed agricultural areas, and overgrazed fields in permanent preservation areas [107]. The concentrations of PTEs (e.g., Cu, Ni, As, Cr, Zn, and Pb) observed in the analysis can cause adverse effects through bioaccumulation in biota tissues and affect the distribution and density of benthic organisms, altering the composition and diversity of communities [107].

The various mining projects planned for Rio Grande do Sul, such as the exploration of phosphate in Lavras do Sul [108] and Ti and other minerals in São José do Norte [109], have great potential to pollute ecosystems with mining waste containing various ETPs, threatening food security and causing harm to the health of humans and animals. Many of these projects have the broad support of the government of the State of Rio Grande do Sul [108,110].

Finally, it is important to highlight that climate change and extreme weather events have the potential to remobilize many PTEs in the environment. For example, heavy rainfall facilitates the migration of PTEs into the environment. The mobilization of PTEs causes important changes in their distribution in soil, air, and water, thus exposing plant and animal communities to new mixtures and quantities of PTEs, with long-term detrimental impacts on flora and fauna [111].

## 7. Atmospheric Pollution

### 7.1. Impacts on Humans

Atmospheric pollution is a major risk factor for non-communicable diseases in Rio Grande do Sul [112]. In a study performed by Agudelo-Castañeda et al. [113] in the metropolitan area of Porto Alegre, atmospheric PAHs with high molecular weight (i.e., benzo[*a*]anthracene, chrysene, benzo[*b+k*]fluoranthene, benzo[*a*]pyrene, indeno [1,2,3-*cd*]pyrene, dibenz[*a,h*]anthracene, and benzo[*ghi*]perylene) were associated with respiratory and cardiovascular diseases. On the other hand, the relationship between air pollutants and viral respiratory infections may be more complex and variable. Also in Porto Alegre, an increased prevalence of severe acute respiratory infections (SARIs) was significantly associated with a period of higher temperatures, but lower concentrations of air pollutants [114]. One possible explanation for this finding is that this period of higher temperatures was also associated with more rain, increasing the risk of infection due to weather conditions and at the same time contributing to the reduction in air pollution [114].

Despite ongoing issues, air quality in the metropolitan area of Porto Alegre has historically improved concerning particulate matter. A recent study [115] showed that air pollution levels (PM_10_ and PM_2.5_) in this area decreased by more than 45% between 2002 and 2021, with significant benefits in terms of improvements in human health (i.e., reduction in air pollution-related deaths). On the other hand, ozone (O_3_) levels increased significantly in the same period [115]. In other words, despite some positive results in relation to particulate matter levels, efforts are still needed to ensure that the air quality in the metropolitan region of Porto Alegre is suitable for human health.

Workers exposed to traffic-related air pollution undergo a series of pathogenic molecular changes that can trigger various diseases [116]. A set of studies carried out with taxi drivers in Porto Alegre found associations between exposure to air pollution and increased risks of atherogenesis and carcinogenic processes, impaired immune regulation, higher levels of inflammation, oxidative stress, and DNA damage. These results were associated with the toxic effects of co-exposure to multiple air pollutants, including particulate matter, PAHs, and PTEs such as Hg [117,118,119,120,121].

Air pollutants can indeed show genotoxic, carcinogenic, and mutagenic activities [113,122]. Fleck et al. [123] reported that high concentrations of air pollutants [i.e., ozone (O_3_) and nitrogen dioxide (NO_2_)] in Porto Alegre, especially in areas of higher population density, were associated with genotoxicity markers in humans (micronucleus test) and plants (pollen abortion assay with *Bauhinia variegata*). A study performed in Santo Antônio da Patrulha City showed that air pollutants (PM_2.5_) potentially related to local vehicular emissions have mutagenic activity [124]. In agreement, other studies reported that the air from the cities of Porto Alegre, Montenegro, and Santo Antônio da Patrulha indeed showed mutagenic and genotoxic potential due to the presence of PTEs, nitro-aromatic compounds, and PAHs (i.e., benzo[*ghi*]perylene, indeno [1,2,3*-cd*]pyrene), among other non-characterized pollutants [125,126,127].

According to data collected from humans living in Rio Grande do Sul’s cities with coal mining activities (Aceguá, Pinheiro Machado, and Candiota), environmental exposure to mineral coal and by-products was associated with increased serum levels of tumor necrosis factor-α (TNF-α), an important inflammatory marker [128]. Of note, the city of Candiota is a major mining site in Rio Grande do Sul, with 46% of its GDP linked to mining activities (e.g., coal and limestone) and coal-burning power generation [129]. These activities contribute to the poor air quality observed in the city. Notably, low levels of PTEs, including Pb, As, Cd, Se, and Ni, are found in Candiota’s PM_10_ samples [130]. However, it is important to highlight that there are no safe levels of some PTEs, such as Pb, when they reach and accumulate in an organism [74,75], making even low levels harmful to human health. Individuals from Candiota showed a 20% higher risk of altered pulmonary function [131], and at least 11% of deaths from cardiovascular problems observed in Candiota’s region are attributable to atmospheric pollution by PM_2.5_ [132].

Volatile organic compounds in the external environment were found at a 22% higher concentration in an urban school in the city of Canoas (Porto Alegre’s metropolitan region) compared to a rural school in Nova Santa Rita City [133]. According to a study performed in the Rio Grande, people living in neighborhoods closer to industries are more susceptible to air pollutants and are at increased risk of pollution-related diseases, while children are the segment of the population most affected by the health effects of pollution [134]. In agreement, a non-statistically significant increase in lung changes was observed among children living in a Rio Grande area with higher pollution levels compared to those living in a less polluted area [135].

Analysis of samples of attic dust and air from Triunfo, a Rio Grande do Sul city housing a deactivated wood treatment plant and a petrochemical complex, showed environmental contamination by particulate matter, pentachlorophenol, PAHs, As, Cr, and Cu. This represents genotoxic and health risks for the population living in the city [136,137]. Notably, although people living closer to the petrochemical area are most affected, those living in regions even further away from the pollutant source (i.e., 35km) were also at genotoxic risk [137].

### 7.2. Impacts on Biodiversity

Morphometric, chemical, and genetic changes in plant species can be used as bioindicators of air pollution [138,139,140], as some plants can undergo adaptations to thrive in polluted environments. In a study that evaluated *Microgramma squamulose* in an urban area (Estância Velha City) and a rural site (Novo Hamburgo City) of Rio Grande do Sul, Rocha et al. [138] observed that the hypodermis thickness and the stomatal density of fertile *M. squamulose* leaves were greater in the area with more pollution (urban) compared to a less polluted area (rural). Analyses of ryegrass (*Lolium multiflorum*) leaves collected near the Arroio Schmidt (Schmidt Stream, Sinos River Basin, Campo Bom City) and other cities in the metropolitan region of Porto Alegre showed the presence of high levels of PTEs of major health and environmental concern, especially Pb, Zn, and Cr, indicating high levels of atmospheric pollution in the regions evaluated [139,141]. Increased levels of chromosomal damage, measured by micronuclei formation, were observed in *Tradescantia pallida* exposed to air pollution from urban areas of the Sinos River Basin compared to samples exposed to the air of riparian areas [140].

In addition to plants, other biological models have already demonstrated pollution in cities of the Sinos River Basin. The somatic mutation and recombination test (SMART) in *Drosophila melanogaster* indicated genotoxic activity of air pollutants (PM_10_ and total suspended particulates) sampled at Canoas City [142]. In addition to pollution from industrial activities and the combustion of fossil fuels, analysis of the chemical composition of the Sinos River Basin’s rainwater indicated that agricultural and livestock activities contribute to pollution in this region [143].

Multiple aspects of lichen behavior and models can be used to assess air quality and potential effects on biodiversity, as shown by a study performed in seven cities of Rio Grande do Sul, specifically Esteio, Triunfo, Charqueadas, Montenegro, Santo Antônio da Patrulha, Caraá, and Maquiné [144]. Notably, increased urbanization can cause decreased lichen diversity and vitality [144], higher frequency of lichens with chlorococcoid algae, foliose narrow-lobed thalli, thallus pruina, and soredia as the main reproductive strategy, among other effects associated with urban areas [145]. Additional studies performed across various cities in Rio Grande do Sul have further demonstrated that lichens serve as valuable indicators of air pollution [82,127,145,146,147]. For example, air from Porto Alegre caused morphophysiological damage (e.g., dead and plasmolyzed cells) to *Parmotrema tinctorum* and *Teloschistes exilis* lichens, potentially due to the presence of atmospheric PTEs and PAHs [127]. Finally, a study performed in Canoas indicated that *Canoparmelia texana*, *Dirinaria picta,* and *Punctelia graminicola* are lichen species commonly found in areas of greater anthropic activity. Those species usually occupy the space left by sensitive species that succumb due to the toxic effects of pollution [146]. This exemplifies how pollution can act on biodiversity as an important selective pressure.

## 8. Plastic Pollution

### 8.1. Impacts on Humans

Plastic is an intentionally persistent material that is ubiquitous throughout the global biosphere [148]. Multiple chemical additives (pigments, antimicrobial agents, and heat stabilizers, among others) are added to plastics during their production aiming to provide different functions and characteristics to the material [149]. During the production or disposal of plastics, these additives can be dispersed and bioaccumulated in the environment, contaminating plants, animals and humans. In addition, microplastics in the environment aggregate other pollutants, such as persistent organic pollutants (POPs) and PTEs, which have high affinity with plastic [149,150].

Furthermore, plastic debris, from both macro and microplastics, creates microenvironments conducive to the development of a variety of microorganisms, including pathogenic viruses and bacteria. This “plastisphere” is easily transported in the environment, facilitating the spread of pathogens among humans, animals, and ecosystems, thus increasing the risk of infectious disease [151,152].

Humans can be contaminated with microplastics in different ways, including inhalation [151], direct ingestion [151], and trophic transfer through the consumption of contaminated animals [149]. The effects of microplastics on humans are not well known, but it has been observed that the smallest particles can pass through varied organs and tissues, including the placenta and the blood–brain barrier [149,153]. Absorption of microplastics through several pathways may be responsible for physiological toxicity [153], generating oxidative stress, unbalanced cytokine secretion, cellular damage, DNA damage, and immunological and inflammatory reactions [151,153]. The health effects of microplastics aggregated by chemical compounds and biofilms, as well as their bioaccumulation in human tissues, still need to be further studied.

The southern region of Brazil is characterized by significant levels of microplastic pollution [154]. Specifically, Rio Grande do Sul is home to one of the five cities in Brazil exhibiting the highest density of microplastics per square meter [154]. Furthermore, the Guaíba Lake, located in the metropolitan region of Porto Alegre, is severely affected by various forms of pollution, including plastics and microplastics originating from incorrectly discarded personal-use plastics and industrial waste [48]. Of note, the Guaíba Lake, a crucial cultural, social, and tourism asset for the capital Porto Alegre [52], is concurrently subjected to the inflow of diverse waste types while, at the same time, also serving as a source of drinking water for the local population [48,52]. Furthermore, the Guaíba Lake connects to the Patos Lagoon, which in turn flows into the sea. Consequently, Rio Grande do Sul’s urban pollution is directly linked to the water pollution of coastal cities.

The data regarding environmental pollution by microplastics throughout Rio Grande do Sul indicate alarming trends concerning the potential impacts of this ecological degradation on human health, as detailed previously. However, there is a lack of studies assessing microplastic contamination specifically among individuals residing in Rio Grande do Sul, thereby underscoring the necessity for further research in this domain.

### 8.2. Impacts on Biodiversity

Plastic pollution has emerged as a critical environmental issue with multiple consequences for biodiversity. In Rio Grande do Sul, the coastal region is significantly affected by plastic contaminants originating from various anthropogenic sources, including industrial discharge, fishing gear, improperly disposed waste on beaches, port activities, sewage effluents, and materials transported by the Patos Lagoon into the marine environment [49,155]. Plastic derived from anthropogenic activities has become one of the main components of marine debris, which poses a threat to marine ecosystems [156].

The coastal region of Rio Grande do Sul serves as a significant habitat for both native fauna and seasonal migratory species [46]. This ecosystem is severely affected by plastic pollution originating from terrestrial sources, such as litter deposited on beaches, and marine pollution. Additionally, the hydrological connectivity with the Patos Lagoon facilitates the direct influx of urban waste from various water bodies within the state, exacerbating the pollution in the coastal environment. As mentioned previously, the southern region of Brazil is severely affected by microplastic pollution [154].

Green turtles (*Chelonia mydas*) and various seabird species seasonally utilize the Rio Grande do Sul’s coastal region for foraging, growth, and migratory activities [46]. An investigation into the ingestion of synthetic debris by these taxa, conducted through the examination of the gastrointestinal tracts of stranded carcasses along an extensive (~350 km) stretch of the southern coastal region of the state, revealed debris contamination in 100% of the green turtles and in 40% of seabird specimens from multiple species [46]. Notably, plastics predominantly constituted the debris identified [46]. Another analysis of green turtle carcasses, collected between the beaches of Balneário Pinhal and Mostardas, in the mid-coast of Rio Grande do Sul, indicated that 88% of the specimens had ingested synthetic debris [156].

Plastic pollution has been observed in Rio Grande do Sul and has affected its biodiversity for many years. In 2001, analyses of the carcasses of green turtles revealed the presence of plastic in the esophagus and stomach of 60.5% of the individuals, which were collected along various beaches on the state’s coastline [157]. Additionally, carcasses of loggerhead turtles (*Caretta caretta*) and leatherback turtles (*Dermochelys coriacea*) provided further evidence of plastic contamination and anthropogenic impacts on these species [157]. In 2009, investigations of both carcasses and live specimens of birds, specifically *Macronectes giganteus*, found that nearly 87.5% of the individuals had ingested debris [158].

Environmental analyses conducted in the coastal region of northern Rio Grande do Sul indicate a notable predominance of plastic as a contaminating agent. Sand samples collected from the dunes and beaches of Xangri-lá revealed a significant increase in plastic pollution during February, a month characterized by heightened anthropogenic activity in the area [155]. Similarly, Praia Grande, a beach also located in the northern part of the state, exhibited substantial pollution by microplastics [159]. In the quantification of debris, polyethylene and polypropylene were the most frequently identified materials. These findings were expected, given that polyethylene and polypropylene rank among the most widely produced plastics globally and are utilized across a variety of applications [159]. Additionally, polyamide was frequently detected, resulting from the strong economic trend of fishing in the region, as polyamide is commonly used in the production of fishing lines. Areas adjacent to intense fishing activity demonstrated heightened pollution levels [159].

In urban areas, the prevalence of materials associated with plastic pollution differs from that observed in coastal regions. The Sinos River has a densely populated and industrialized basin [160]. This river flows through several cities in Rio Grande do Sul and supplies a significant portion of the state population. However, it also receives substantial amounts of pollution and untreated sewage [160]. According to the Brazilian Sanitation Ranking (Ranking do Saneamento) conducted by the Trata Brasil Institute (Instituto Trata Brasil), two cities along the Sinos River, namely Canoas and Gravataí, are listed among the 20 municipalities with the poorest sanitation over the past decade [161]. Poor sanitation facilitates the infection of wildlife with pathogens, harming the reproduction, well-being, and survival of different species [162]. The association between plastic debris and microorganisms, the plastisphere, can exacerbate this problem.

An analysis of plastic pollution in the Sinos River revealed that both the river water and drinking water supplied from this source are contaminated by microplastics [160]. The highest concentrations of microplastic pollution were detected in the headwater regions and in urbanized areas, where significant amounts of untreated sewage are discharged. Conversely, the lowest concentrations were recorded in rural areas with minimal human activity [160]. Among the materials identified, approximately 80% were fibers, found in both the river water and drinking water samples. These fibers were likely remnants from synthetic clothing, suggesting that their presence may result from the washing of such garments and their subsequent dispersal through untreated sewage systems [160].

The Sinos River, along with the Jacuí River (Rio Jacuí), Caí River (Rio Caí) and Gravataí River (Rio Gravataí), ultimately flows into the Guaíba Lake, which, in turn, flows into the Patos Lagoon. The Guaíba Lake is highly polluted and serves as a crucial water source for the population of the state capital, while concurrently receiving both treated and untreated sewage [52]. Investigations conducted at various sampling points within the Guaíba Lake revealed microplastic pollution in 100% of the analyses performed [47]. The predominant materials identified in the waste were polypropylene and polyethylene. Furthermore, spectroscopy analyses indicated that the microplastics present in the lake have been persistent for an extended duration [47]. Similarly, the Patos Lagoon also exhibited contamination by microplastics, which were primarily composed of high-density polyethylene, low-density polyethylene, and polytetrafluoroethylene [163]. These findings underscore the pervasive nature of microplastic pollution within these aquatic systems and raise concerns regarding the implications for water quality and ecosystem health. The impacts of microplastics on the biodiversity of the Guaíba Lake and the Patos Lagoon are poorly understood. However, it is likely that the effects will be deleterious, given the toxicity of multiple constituents of microplastics, as discussed previously.

## 9. Pesticide Pollution

### 9.1. Impacts on Humans

Current agricultural production systems make extensive use of fertilizers and pesticides, as these substances significantly increase production. However, their formulations pose a concern for both the environment and public health. Studies conducted in agricultural regions, such as Rio Grande do Sul, have revealed the presence of pesticides such as atrazine, chlorpyrifos, and imidacloprid in drinking water. These contaminants, which are often not removed in water treatment processes, can cause harmful effects on health. Furthermore, in addition to large producers, some groups of small farmers use pesticides intensively, contributing to the contamination of drinking water sources [164,165].

The use of pesticides can result in pollution of surface and groundwater, which has serious impacts on human health. Chemical residues present in drinking water are associated with an increased risk of diabetes, reproductive disorders, neurological dysfunction, cancer in children and adults, and respiratory problems [166]. Analyses of spring waters used for consumption in the northern region of the state, located near areas of pesticide use and forestry activity, did not detect pesticide contamination [166]. However, an analysis of drinking water samples from the São Gonçalo Channel (Canal São Gonçalo) revealed the presence of clomazone, an agricultural herbicide, at concentrations exceeding the established limit in 50% of the samples collected [167]. Residues of pesticides, pharmaceuticals, and personal care products such as atrazine, diuron, propylparaben and mebendazole were identified in treated water in Morro Redondo City in the south of the state of Rio Grande do Sul, highlighting the impact of agricultural activity and domestic sewage on water quality [168]. During the planting season of tobacco, compounds such as chlorpyrifos, imidacloprid, atrazine, simazine, and clomazone were detected in well water and surface streams in the Rio Grande do Sul, demonstrating the risk to human health in areas where water treatment is limited or nonexistent [164]. These results reinforce concerns about agricultural residue and the need for specific and sensitive treatments to eliminate emerging pollutants from water intended for human consumption.

On this matter, Riquinho et al. [169] investigated the relationship between the presence of atrazine in drinking water and mortality from diseases of the genitourinary system. Of the nineteen cities analyzed, three presented atrazine levels equal to or higher than those permitted by Brazilian legislation. A significant positive correlation was observed between atrazine levels and the mortality rate from genitourinary diseases. Atrazine, widely used in corn plantations, has been shown to be overused, indicating potential impacts on human and environmental health [169]. The contamination of drinking water by atrazine and other pollutants reinforces the findings of Pereira et al. [170] regarding the mutagenicity of treated water from areas of intense industrial and agricultural activity.

Consumption of pesticide-contaminated food represents another significant route of exposure. Pesticide residues have been identified in a variety of food products produced in Rio Grande do Sul, including milk [171,172], dairy derivatives [171], honey [173,174], and strawberries [175]. Breast milk samples collected in different regions of Rio Grande do Sul also showed the presence of pesticide residues [176,177]. Continued exposure to these pollutants increases the burden of oxidative stress induced by pesticide toxicity, impairing DNA repair mechanisms and contributing to the development of several diseases [178]. Furthermore, organochlorine pesticides have high environmental persistence and biomagnification along the food chain, being especially dangerous for children due to their impact on cognitive development and the immune system [172,174].

Furthermore, Rodrigues et al. [179] detected pesticide residues in honey samples from stingless bees (Meliponinae) collected in northern Rio Grande do Sul. Pesticides used in nearby plantations, such as soybeans and corn, contaminated floral sources, resulting in varying pesticide concentrations between bee species [179]. This contamination reflects the indirect impact of intensive agriculture on biodiversity and food security. Cheeses produced in Rio Grande do Sul also show contamination by pesticides. Residues of organochlorine compounds, including dichlorodiphenyltrichloroethane (DDT) and aldrin were detected in samples of both industrialized and artisanal cheese [180].

In Rio Grande do Sul, family farmers have been continuously exposed to multiple pesticides due to a lack of technical guidance, inadequate use of personal protective equipment, and low awareness of the associated risks. Sasso et al. [181] evidenced biochemical and immunological alterations in these workers, including increased levels of lipid peroxidation and carbonyl proteins, reduced glutathione levels, and glutathione reductase activity, as well as alterations in inflammatory responses [181]. These alterations compromise the body’s defense mechanisms, increasing vulnerability to chronic diseases and acute poisoning. In addition, workers exposed to pesticides face genotoxic risks. Floriculturists in southern Brazil, who frequently handle pesticides without adequate protection, present high rates of DNA damage, as evidenced by cytogenetic tests [182]. A cross-sectional study identified a correlation between pesticide use and the prevalence of chronic diseases in the Rio Grande do Sul’s rural population, with emphasis on neurological and oral disorders [183]. These data highlight the importance of using protective equipment and strict regulation in the use of these compounds.

A study conducted in Santa Cruz do Sul City revealed that chronic exposure to pesticides and nicotine (present in tobacco leaves) is associated with cellular damage, as evidenced by abnormal shortening of telomeres in exposed farmers. Glyphosate stood out as the most prevalent pesticide, being used by 92% of the exposed individuals, followed by flumetralin, used by 28.5% of them. Notably, exposure to mixtures of pesticides was significantly associated with reductions in telomere length. Abnormal telomere shortening can accelerate cellular aging and is a known risk factor for several chronic diseases, including cancer and cardiovascular disease. Thus, the study reinforces the need for strict control over pesticide use, considering the significant impacts on human health, especially in highly exposed rural communities [184].

### 9.2. Impacts on Biodiversity

Pesticides also cause significant impacts on several animal species, both through direct contact and through bioaccumulation in ecosystems. A robust body of evidence shows that pesticide residues are found in soils, rivers, surface water, and freshwater in different regions of Rio Grande do Sul [35,164,165,166,167,185,186,187,188]. The quantity and diversity of toxins used vary according to the type of crop and the period of planting. Contaminated areas are primarily a result of soybean, rice, wheat, corn, and tobacco cultivation in the region. It is well-established that biodiversity is significantly impacted by this agricultural model, whether through land use changes, anthropogenic activities, or both direct and indirect pesticide contamination [36,189].

Do Amaral et al. [165] investigated the presence of pesticides in specimens of fiddlehead (*Loricariichthys anus*) and acará (*Geophagus brasiliensis*), both fish found in the Jacuí River. The authors detected the presence of pesticides such as atrazine, simazine, azoxystrobin and imidacloprid in the fish samples, in addition to behavioral changes and damage to muscle and nerve tissue. Notably, identifying the direct impacts of these contaminants is complex, given that environmental factors, such as water pH and temperature, also play an important role [165]. Marinowic et al. [190] demonstrated significant DNA damage in *Bryconamericus iheringii* fish exposed to β-cyfluthrin, indicating the genotoxicity of pyrethroids. This compound is a commercial insecticide that promotes oxidative stress in exposed organisms, causing irreversible cellular damage that can compromise biodiversity [190].

The bioaccumulation of pesticides in birds of prey, such as nocturnal owls, is an example of the serious consequences of pesticide use for local biodiversity. Compounds such as chlorpyrifos ethyl, detected in the livers of birds collected in Passo Fundo City, interfere with essential enzymatic processes, impacting populations and food chains. As they are bioindicators, their presence highlights the severity of bioaccumulation in agricultural areas of the state [105].

Bees also play a crucial role as bioindicators. Pollinating insects are particularly vulnerable due to their direct interaction with the agricultural environment. Pesticides present in pollen, nectar, and water compromise their health, reducing colonies and contaminating bee products such as honey. This impact directly affects biodiversity and agricultural productivity, in addition to indicating the presence of contaminants in areas relatively far from industrial activity [174]. Pesticides such as the insecticide fipronil have caused the deaths of bee populations in multiples cities in Rio Grande do Sul [191,192].

Amphibians, which are essential for ecological balance, are highly vulnerable to pesticides. Species such as *Pseudis minuta*, which live in aquatic environments, may suffer from behavioral changes, teratogenic and genotoxic effects, and histological lesions caused by herbicides such as glyphosate, widely used in Brazil [193]. Reduction in amphibian populations can lead to an increase in the number of insects, such as mosquitos, that transmit dengue, zika, oropouche, and malaria, impacting both public health and ecosystems [193].

Inadequate pesticide management, combined with a lack of effective public policies, compromises both the health of agricultural workers and the integrity of ecosystems. Therefore, it is urgent to implement integrated sustainable management strategies, strict regulation, and continuous monitoring to mitigate these impacts. Finally, we highlight that a more in-depth discussion on the effects of pesticide pollution in the Rio Grande do Sul’s Pampa biome can be found in a previous study performed recently by our research group [36].

## 10. Extreme Weather Events

### 10.1. Impacts on Humans: Enphasis on Pathogen Pollution

**Figure 6:**
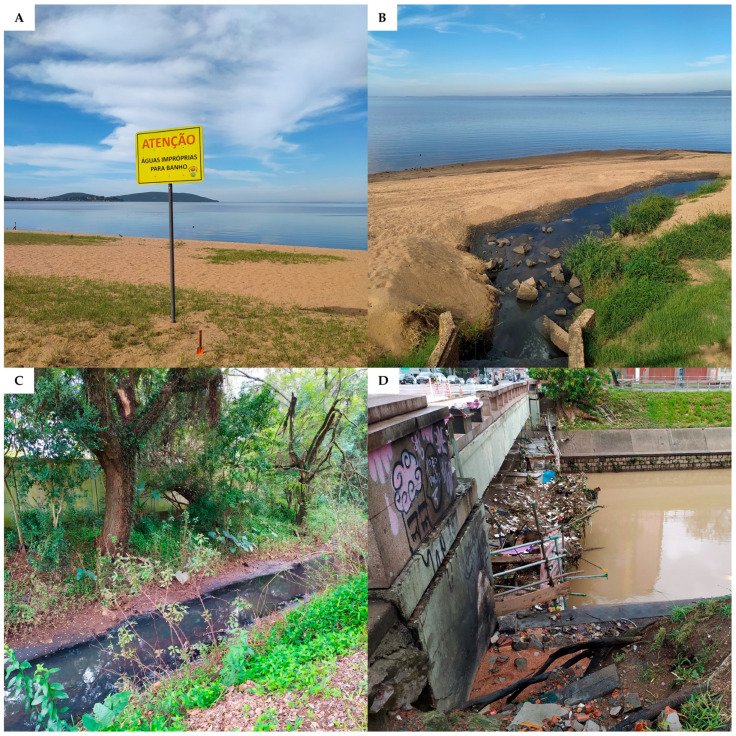
Sanitation-related problems observed in Porto Alegre City. A and C: Ipanema Beach (freshwater beach) in Porto Alegre showing a sign indicating that the water is unfit for swimming during the 2023 summer season (**A**) and the release of domestic sewage into the water at the beach (**B**). (**C**) Presence of domestic sewage in a stream located in a public park in Porto Alegre. (**D**) Presence of accumulated garbage in a bridge repair structure located in Dilúvio Stream, which flows into the Guaíba Lake. The Dilúvio Stream is a habitat for varied fauna, but it presents several classes of pollutants, including toxic metals and biological contamination, thus fueling pathogen pollution and other health issues that affect humans and animals (photo credits: Joel H. Ellwanger).

The Anthropocene is marked by the combined effects of climate change, pollution, and biodiversity loss, which trigger a series of detrimental impacts on ecosystems and human health [194]. Extreme weather events can have their effects on public health magnified when combined with water contamination by pathogens, a frequent problem in Rio Grande do Sul. Figure 6 shows representative images of sanitation-related issues observed in Porto Alegre City.

A study performed with water samples from the São Lourenço River (Rio São Lourenço), which flows into the Patos Lagoon, indicated microbiological contamination by coliforms in more than 90% of the samples analyzed, an alarming result caused by pollution by untreated agricultural and domestic effluents [195].

Between 2018 and 2021, 24,385 hospitalizations and 501 deaths due to waterborne and foodborne diseases were recorded in Rio Grande do Sul [196], evidencing the deficiencies in the state’s sanitation systems. Pathogen pollution, in addition to affecting the biodiversity of aquatic and terrestrial ecosystems [162], becomes particularly harmful to human health in situations of extreme climate events, such as floods that facilitate the exposure of human populations to zoonoses and water-borne pathogens, as was observed in a mega flood that hit Rio Grande do Sul in 2024 [197,198]. More than 700 cases of leptospirosis were triggered by this flood in the state [198]. Similarly, a ~300% increase (above the monthly average) in hepatitis A cases was observed in Encantado City, Taquari Valley, after a flooding event occurred in 2013 [199]. Beyond infectious diseases, extreme weather events like floods also facilitate the occurrence of mental problems (e.g., depression, anxiety, and trauma) which are associated with immunosuppression, physical injuries, pulmonary diseases, and exposure to toxic substances, as well as intensifying the burden of chronic diseases [64,197].

In Rio Grande do Sul, similarly to different places around the world, climate disasters affect territories and populations unequally, with the same type of extreme weather event leading to different impacts depending on the socio-spatial conditions of the affected location [200]. Climate disasters have a greater impact on socially vulnerable populations which already had deficiencies in housing and sanitation conditions before the disaster occurred. In the southern region of Brazil, projections of an increase in extreme rainfall events combined with high population density and precarious urbanization areas tend to increase the risks related to climate change in the region [201]. In this sense, the expansion and improvement of sanitation systems in Rio Grande do Sul are essential to limit pathogen pollution and the spread of infectious diseases during future extreme weather events.

### 10.2. Impacts on Biodiversity

Different ecosystems exist in Rio Grande do Sul. The Taim Ecological Station (Estação Ecológica do Taim), for example, is located between the cities of Rio Grande and Santa Vitória do Palmar, on the Coastal Plain of Rio Grande do Sul. Areas of Taim occupied by native grassland and close to roads are more susceptible to fires [202]. Studies performed in the South Brazilian highland grasslands showed that fire, depending on intensity and specific conditions (i.e., prescribed patch burnings, with well-defined intervals), can contribute to the biodiversity of grassland ecosystems, as it may create a biological heterogeneity, forming multiple ecological niches for different species of birds, for example [203,204]. However, uncontrolled, anthropogenic fires are a threat to Taim’s biodiversity [202]. Two major fires were indeed recorded in Taim in 2008 and 2013 [205], affecting the flora and fauna of this ecologically sensitive region, with effects ranging from species mortality to forced changes in behavior [206].

Factors related to climate change, such as increased temperature and decreased canopy cover, can affect wild primates in Rio Grande do Sul, such as *Alouatta* populations. These factors can modify the arboreal habits of primates, forcing more ground use and thus causing changes in their behavior, diet, and susceptibility to predators [207]. Climate change is also a major cause of amphibian decline in Rio Grande do Sul’s ecosystems due to the loss of territory with a climate suitable for some species, including *Melanophryniscus cambaraensis* and *M. tumifrons* [208], and climate-induced alterations in reproductive success. For instance, periods of extreme drought can desiccate frog eggs [209].

Droughts can create favorable climatic conditions for the spread of toxic diseases among animals. A drought observed in 2005 favored the development of the hymenopteran insect *Perreyia flavipes* (greater viability of larvae in cocoons), causing three outbreaks of *P. flavipes* larvae poisoning in cattle in southern Rio Grande do Sul in 2006. Poisoning may occur through accidental or intentional ingestion of larvae by cattle along with vegetation [210]. On the other hand, an increase in the frequency of extreme rainfall events is also expected as climate change intensifies. Notably, heavy rains intensify the sandization process in Rio Grande do Sul [211], causing important ecosystem changes with overlooked impacts on fauna and flora.

The Patos Lagoon basin is a climate change-sensitive ecosystem, especially concerning water availability. Climate change may cause a significant increase in water discharge into the ecosystem [212]. This can cause changes in the composition of the water (e.g., pH, salinity, amount of nutrients, sediments, and pollutants), negatively affecting aquatic species. This was observed during the 2024 mega flood in Rio Grande do Sul, when a large amount of flood water and sediment reached the Patos Lagoon [213,214]. In summary, climate change combined with pollution and other anthropogenic activities like overfishing will have an increasingly detrimental impact on fish communities in the Patos Lagoon basin [215].

## 11. Perspectives

A wide variety of pollutants (e.g., plastics, pathogens, and PTEs) reach ecosystems as a result of inefficient waste management in urban environments and insufficient sewage collection and treatment systems observed in many cities in Rio Grande do Sul, such as the capital Porto Alegre [52]. In this sense, the adequate implementation of the National Solid Waste Policy (Política Nacional de Resíduos Sólidos), which includes the State Solid Waste Plans (Planos Estaduais de Resíduos Sólidos) [216,217], and the expansion of sanitation infrastructure are fundamental actions to solve pollution-related problems in Rio Grande do Sul.

Especially regarding plastic pollution, Brazil should consider banning single-use plastics, as other countries such as Rwanda already have [218]. Policies that make the plastic producers pay for waste management and recycling, as already observed in Spain, are required [219]. Also, new technologies for recycling plastic, similar to highly efficient solutions already in place in England [220], are urgently needed. However, above all these initiatives, the most crucial step is to reduce plastic demand. This requires substantial changes in traditional industrial production, which heavily relies on plastics, as well as active public participation. Additionally, stronger government policies must be implemented to minimize plastic usage.

Concerning air pollution, Brazil recently instituted the National Air Quality Policy (Política Nacional de Qualidade do Ar—PNQAr), which represents a step forward in the country’s intention to better monitor air quality throughout the country, along with clear objectives to prevent and reduce air pollution, also defining responsibilities for state governments [221,222]. The PNQAr defines that state environmental agencies must prepare a State Air Quality Management Plan within a maximum period of two years after the publication of the state inventory of atmospheric pollutant emissions [221]. Of note, the National Environment Council (Conselho Nacional do Meio Ambiente—CONAMA) defines specific limit values for the quantity of various atmospheric pollutants [particulate matter (PM_10_), particulate matter (PM_2.5_), sulfur dioxide (SO_2_), nitrogen dioxide (NO_2_), ozone (O_3_), smoke, carbon monoxide (CO), total suspended particles, and Pb] valid for Brazil in 2024 and future years (i.e., 2025, 2033, 2044, and beyond) [222,223]. Examples from other countries show that solutions to improve air quality involve measures such as improving public transport, preventing waste burning and fires, creating air quality authorities, and establishing an efficient air quality tracking system [224].

Regarding climate change, nature-based solutions for climate change mitigation with the potential to create environmental and socio-economic benefits involve avoiding forest conversion and coastal impacts, reforestation, natural forest management, developing sustainable agricultural practices, restoration of coastal regions and wetlands, among others [225]. However, global problems require local solutions. In this sense, it is the responsibility of the Government of the State of Rio Grande do Sul to develop concrete actions to combat climate change and reduce the impacts of extreme weather events [226]. The Climate Advisory Office (Assessoria do Clima) linked to the Secretariat of Environment and Infrastructure (Secretaria do Meio Ambiente e Infraestrutura) of the State of Rio Grande do Sul released in 2023 a set of strategies for tackling climate change (ProClima2050) focused on climate resilience, fair energy transition, reduction of greenhouse gas emissions, and environmental education and awareness [227]. Although many of the activities foreseen in this plan are welcome, in practice, several aspects of the Rio Grande do Sul’s environmental agenda are misguided and business focused. For example, energy transition is important and seen as the solution to climate problems in Rio Grande do Sul (energy production with lower greenhouse gas emissions, such as wind and solar power). However, it does not address the root of the problems related to pollution and climate crisis, which are land-use changes and the intensive demand for raw materials and energy, with the consequent production of waste and atmospheric pollution with greenhouse gases. Energy transition projects under a “sustainable” discourse have been used in Rio Grande do Sul to favor a neoliberal agenda to generate profits for “green energy” companies operating in the state. In this sense, Rio Grande do Sul’s government has neglected the environmental and social impacts of wind power generation projects and has supported mining projects in ecologically sensitive regions of the state [110].

Economic growth is not infinite and therefore curbing the production of items that are dispensable for life on Earth is an urgent need, especially considering that the Earth’s capacity to receive anthropogenic waste (pollution) is finite. Exceeding this capacity makes pollution a threat to the lives of humans and animals. The quest for infinite economic growth, symbolized by the obsession with ever-increasing GDP percentages, is incompatible with a planet with finite natural resources [228]. Governments and societies must move away from the obsession with infinite economic growth, which leads to excessive consumption and fosters artificially created demands aimed at advancing a capitalist vision. Alternatively, human societies should focus on producing high-quality, durable products and promoting services and jobs that genuinely contribute to social equality within a framework known as degrowth [229], prioritizing human and animal well-being and long-term ecological sustainability over short-term growth based on an economy supported by high energy demands from fossil fuels.

The environmental debate in Brazil, particularly regarding the impacts of agriculture and the current economic system, often centers on the Amazon rainforest. While combating Amazon degradation is critical for addressing global climate change and its repercussions across Brazil, it is equally important for Brazilian environmental policies to expand their focus to include non-forest biomes such as the Pampa. The ecosystems in Brazil’s southern region provide essential ecosystem services and require stronger conservation efforts to ensure their preservation.

Solutions for the preservation of the Pampa must be formulated considering the specific characteristics of this biome. For example, grazing can reduce wildfire risk in this ecosystem which is dominated by grassy vegetation, since grazing animals can reduce dead herbaceous vegetation in addition to making other changes to the vegetation that reduce the risk of uncontrolled fires [230]. Also, measures such as combating hunting, developing sustainable and pesticide-free agricultural and livestock practices, protecting indigenous territories, and expanding legally protected areas are fundamental to preserving the Brazilian Pampa [37].

**Figure 7:**
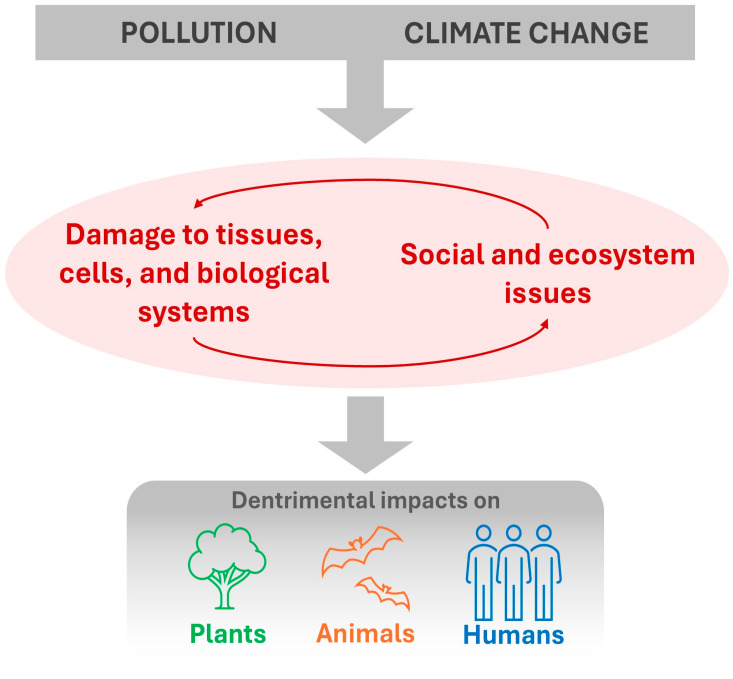
Combined consequences of pollution and climate change.

In summary, the environmental challenges faced by Rio Grande do Sul add to the broader environmental crises impacting other Brazilian biomes, such as deforestation in the Amazon and wildfires in the Pantanal and Cerrado. A development model driven by the exploitation of natural resources and agriculture for the profit of a few, coupled with reduced public investment in sanitation systems and insufficient measures to address the climate emergency, is exacerbating the pollution and degradation of Rio Grande do Sul’s diverse ecosystems, with detrimental impacts on human and animal health (Figure 7). Actions by civil society and the continuous work of environmental organizations are crucial for raising awareness of environmental issues in Rio Grande do Sul and for ensuring effective oversight of environmental protection efforts by the municipal and state governments.

## 12. Conclusions

**Figure 8:**
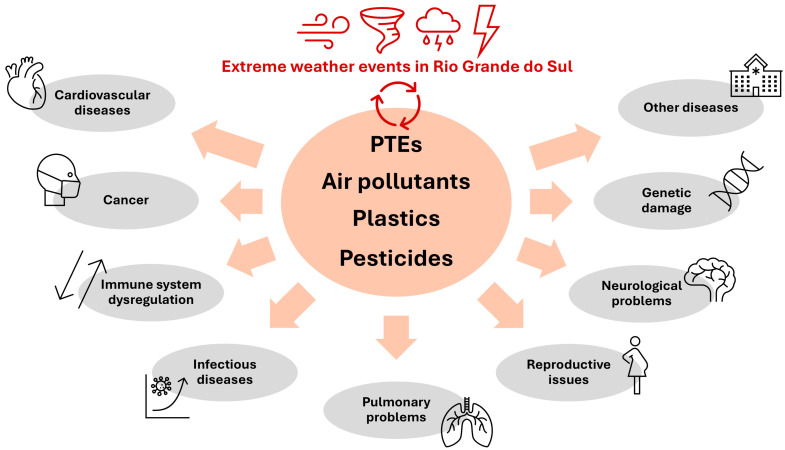
Health problems observed in the human population of Rio Grande do Sul, which may be exacerbated by climate change. PTEs—potentially toxic elements.

This review provides an overview of the environmental situation in Rio Grande do Sul, focusing on the challenges posed by pollution and climate change. Based on the sources reviewed, the following key conclusions can be drawn:

- Two biomes occur in Rio Grande do Sul, the Pampa and the Atlantic Forest. The transition between these two biomes, known as an ecotone zone, associated with the characteristic landscape of each biome, creates landscapes with great biodiversity.
- Pollution intensified in Rio Grande do Sul starting from the colonization of the state by Europeans (especially in the 19th century) and subsequent industrialization process in the second half of the 20th century.
- Historically, Rio Grande do Sul has played an important role in Brazilian environmentalism, being a pioneering state in various awareness and environmental policy actions.
- Currently, activities such as mining, agriculture, industrial tree farming, and unplanned urban expansion in Rio Grande do Sul threaten biodiversity and human health. These threats are exacerbated by climate change and the combined effects of different pollutants.
- Extreme weather events are an increasing threat to Rio Grande do Sul.
- Deficiencies in sanitation systems are observed in different cities of Rio Grande do Sul, including the capital Porto Alegre, facilitating the contamination of the environment with different pathogens and toxic pollutants.
- In 2024, a mega flood hit Rio Grande do Sul, causing immense damage to human health and biodiversity. This flood, associated with pathogenic pollution, caused a major leptospirosis outbreak.
- Agricultural activities in monoculture systems (cash crop plantations such as soybeans, rice, and tobacco) are so intense in Rio Grande do Sul that they place the state among the largest consumers of pesticides in Brazil, contaminating soil, water, and air, and thus harming humans, fauna, and flora.
- Plastic pollution causes visual pollution and threatens the survival of wildlife in Rio Grande do Sul.
- Microplastic pollution is widespread in the different ecosystems of Rio Grande do Sul, posing health risks to different animal species and humans.
- Mining activities in Rio Grande do Sul are important sources of pollution by toxic metals that contaminate soil, air, and water, causing deleterious effects to different cells, organs, and tissues.
- Climate change can exacerbate PTE pollution in the state, as it has the capacity to modify the distribution of these elements in ecosystems, exposing species to new combinations of toxic agents.
- Industrial activities, mining, and the vehicle fleet in Rio Grande do Sul pollute the air with particulate matter that carries different toxic substances, such as metals and organic pollutants.
- Fires in the Pampa and other Brazilian biomes, such as the Amazon Forest and Pantanal, contribute to the atmospheric pollution observed in Rio Grande do Sul.
- Construction activities in Rio Grande do Sul are an important source of solid waste and greenhouse gases that contribute to climate change.
- The plastisphere associated with deficiencies in sanitation systems can facilitate the spread of infectious diseases in Rio Grande do Sul.
- The different classes of pollutants of greatest importance in Rio Grande do Sul (i.e., atmospheric pollutants, toxic metals, plastics, and pesticides) are associated with genetic damage, neurological, pulmonary, reproductive and cardiovascular problems, dysregulation of the immune system, and cancer, among other diseases in humans. Climate change may exacerbate these health problems (Figure 8).

- Pollution associated with climate change is a major driver of biodiversity loss in southern Brazil.
- Improvements in solid waste management and sanitation systems are essential actions to control pollution in Rio Grande do Sul.
- State and municipal environmental protection agencies need to increase monitoring of industrial, mining, and agricultural activities in the state, punishing irregularities efficiently.
- Mining projects in Rio Grande do Sul must be reduced as they show highly polluting potential.
- Air quality monitoring and control in the state needs to be urgently improved.
- Climate change adaptation plans must be implemented in Rio Grande do Sul, associated with policies to reduce greenhouse gas emissions from the state’s agricultural, industrial and livestock activities.
- Environmental agencies, environmental activities, and researchers must intensify the study of the biodiversity of the Pampa biome, in addition to increasing the study of the impacts of pollution and climate change on the rich biodiversity of this often neglected biome.
- Active public participation is crucial to ensuring the effective implementation of environmental protection measures in Rio Grande do Sul, helping to reduce pollution and mitigate climate change.

## Data Availability

No new data were created or analyzed in this study. Data sharing is not applicable to this article.
